# Study on the mechanical properties and failure mechanism of a multi-lithologic layered rock mass

**DOI:** 10.1016/j.heliyon.2024.e41233

**Published:** 2024-12-14

**Authors:** Bo Zeng, Guozhou Qiu, Haoyong Huang, Yintong Guo, Ersi Xu, Junchuan Gui, Junfeng Li

**Affiliations:** a Shale Gas Research Institute of PetroChina Southwest Oil & Gas Field Company, Chengdu, Sichuan, 610051, China; b State Key Laboratory of Geomechanics and Geotechnical Engineering, Institute of Rock and Soil Mechanics, Chinese Academy of Sciences, Wuhan, 430071, China; c School of Pipeline and Civil Engineering, China University of Petroleum (East China), Qingdao, 266580, China

**Keywords:** Multilithology, Rock mass, DIC, Mechanical properties, Failure mechanism, Interface feature

## Abstract

The mechanical properties of multi-lithologic reservoir rock masses are complex, and the failure mechanism is not clear. This research belongs to the field of oil and gas exploration and development. Brazilian splitting, and digital image correlation (DIC) tests were performed to study the mechanical properties and failure mechanism of assemblages containing sandstone, shale, and limestone. In the Brazilian splitting test, the tensile strength of the rock mass first increased and then decreased with an increasing angle. As the interface angle increased, The morphology of the splits is also changing. In the DIC test, according to the deformation characteristics at different angles, the Brazilian splitting test process was summarized into three stages: interface deformation, elastoplastic, and failure, the interface has a great influence on the fracture propagation, and the fracture tends to expand along the interface. Interface characteristics were studied based on 3D scanning technology and fractal method, with an increase in the angle, the fractal dimension D showed first decreased, then increased, and further decreased, which was identical to the variation trend of the maximum load in the Brazilian splitting test and the maximum principal strain in the DIC test with the angle. This study provides reference for predicting fracture propagation trend in field hydraulic fracturing work.

**Table:** Nomenclatures

σ	Normal stress, MPa
τ	Shearing stress, MPa
D	Brazilian splitting specimen diameter, m
l	Brazilian splitting sample thickness, m
(x_refi_, y_refj_) and (x_curi_, y_curj_)	The coordinates of the sub-regions before and after deformation
f_m_ and g_m_	The average gray values of the reference and current images
D	Fractal dimension
δ_i_	The ith time square box count size
N	Number of square boxes
E	Elasticity modulus, GPa
u, v	Displacement, m
S	A setwhich contains all of the subset points.
n(S)	The number of data points in subset S

## 1 Introduction

With the development of oil and gas exploration and development technology, the research focus of oil and gas exploration and development has gradually changed from conventional reservoirs to unconventional reservoirs, and unilithologic reservoirs to multi-lithologic reservoirs. Multi-lithologic reservoirs are unconventional reservoirs with diverse lithologies, substantially different rock mechanical properties, and complex in situ stresses [1], which tend to destabilize the mechanical systems during exploration and exploitation, such as uneven deformation during drilling process and casing deformation caused by shaft wall instability and damage [2]. Many factors affect rock failure [3], including rock anisotropy, geological structure, lithology combination and spatial variation, rock thickness and variation, hydrogeology, and pore pressure distribution [4]. Studying the macroscopic deformation, failure mechanisms, and mechanical properties of multi-lithologic rock assemblages in shale reservoirs is of great significance to ensure safe and efficient oil and gas production [5]. In this study, the mechanical properties and failure mechanisms of multi-lithologic layered assemblages in a shale reservoir were investigated.

In the study of unilithologic stratified rock masses, the profile characteristics of shale (layered rock mass) under uniaxial conditions were studied [6]. The rock exhibited different failure modes with a change in the bedding angle [7]. The effects of the bedding plane meso-parameters on the mechanical properties of the layered rock mass were studied. The results of Xia [8] showed that when the bonding strength did not differ from that of the bedrock, it considerably affected the strength, whereas bedding friction had a weak effect on the strength. The influence of the shale bedding angle on uniaxial tests was studied, and a method for evaluating shale brittleness was established [9]. A hypothesis of progressive coordinated shear failure was proposed by shear tests on limestone, sandstone, and mudstone composite rocks In a study of multi-lithologic layered rock masses [10]. The failure criterion of the composite rock mass was studied based on the assumption that the composite rock mass follows the Mohr–Coulomb strength criterion [11]. Considering a stratified rock mass as a complete mechanical system, a mechanical model of a composite system was established [12]. A modified criterion based on the transverse isotropic body strength has been proposed by Omid et al. [13]. A new formula of relation between c, ϕ, and bedding dip angle has been established [14]. A layered composite rock mass composed of similar materials was tested using uniaxial compression [3]. The results showed that the failure of the vertical layered structure specimens is closely related to the rock strength of each layer but not the interlayered bond strength, whereas the failure of the horizontal layered structure specimens is closely related to the interlayered bond strength [15]. Damably et al. [16] proposed a method to directly measure the shear modulus of transversely isotropic rocks using a uniaxial compression test. Chekhov [17] developed an accurate method for studying the surface instability of regularly layered semi-infinite media to discuss the problem of allowing rocks to exhibit inelastic properties in a stable layered rock mass. Liu [18] proposed an anisotropic seepage stress coupling model of the mechanical behavior of layered rock slopes based on anisotropic elastic theory aimed at the anisotropic characteristics of layered rock masses. Wang [19] established a calculation model considering the elastic anisotropy based on the linear elastic theory and displacement equivalent. The equivalent elastic modulus and equivalent Poisson's ratio of layered rocks perpendicular and parallel to the loading direction are discussed. The relationship between the material properties, geometric parameters, equivalent elastic modulus, and equivalent Poisson's ratio in both directions was studied.

In previous studies, the mechanical properties of rock masses have mainly been studied from the perspective of rock joints, cleavage, foliation, micro- and macro-cracks, and stratification, whereas multi-lithologic layered rock masses have rarely been studied [20]. In this paper, multi-lithologic stratified rock mass was artificially prepared, Brazil splitting test and digital image correlation (DIC) test were conducted at different angles, and the surface characteristics of fractured rock were analyzed based on high-precision fracture topography scanning technology, so as to study the mechanical characteristics and failure mechanism of multi-lithologic stratified rock mass.

## 2 Mechanics and DIC calculation principles

### 2.1 Brazilian splitting stress distribution

**Fig. 1:**
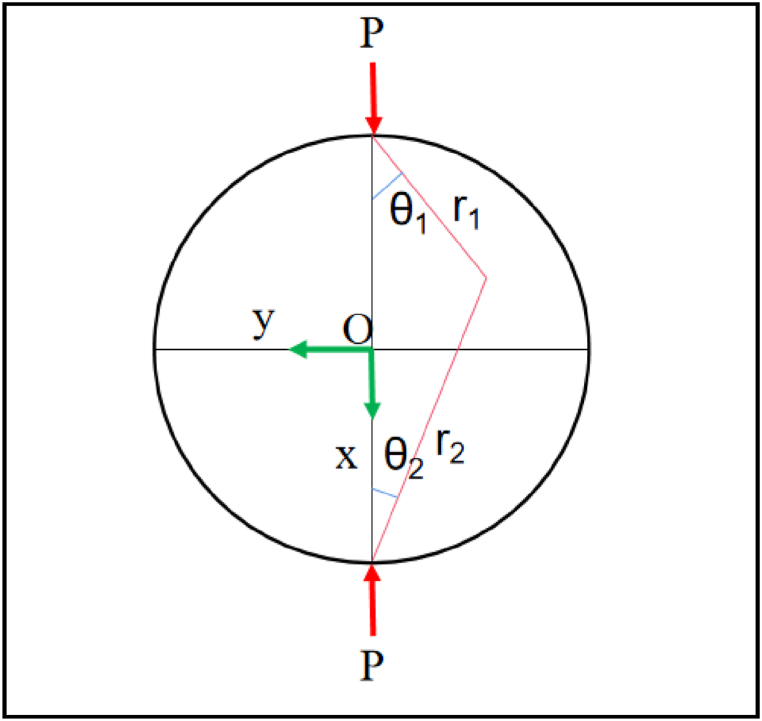
Force analysis diagram of Brazilian splitting plane [21].

Fig. 1 shows the stress distribution in the Brazilian splitting test under ideal conditions.

(1) $$\begin{array}{l} \sigma_{x}=\frac{2P}{\pi l}\left(\frac{\cos^{3}\theta_{1}}{r_{1}}+\frac{\cos^{3}\theta_{2}}{r_{2}}\right)-\frac{2P}{\pi Dl}\\ \sigma_{y}=\frac{2P}{\pi l}\left(\frac{\cos\;\theta_{1}\;\sin^{2}\theta_{1}}{r_{1}}+\frac{\cos\;\theta_{2}\;\sin^{2}\theta_{2}}{r_{2}}\right)-\frac{2P}{\pi Dl}\\ \tau_{xy}=\frac{2P}{\pi l}\left(\frac{\cos^{2}\theta_{2}\;\sin\;\theta_{2}}{r_{2}}+\frac{\cos^{2}\theta_{1}\;\sin\;\theta_{1}}{r_{1}}\right) \end{array}$$

Formula (1) reflects the stress characteristics of the Brazilian disk. When θ_1_ = θ_2_ = 0, r_1_ = r_2_ = D/2, that is, the force state at the origin O is as formula (2) [21].

(2) $$\begin{array}{l} \sigma_{x}=\frac{6P}{\pi Dl}\\ \sigma_{y}=-\frac{2P}{\pi Dl}\\ \tau_{xy}=0 \end{array}$$

### 2.2 Strain monitoring methods

A layer of uniform white paint was sprayed on the sample surface, which detected the strain as the background color, and then the black speckle was sprayed randomly. Strain calculations were performed by reading the position information of the black speckle.

According to Blaber et al. [22], the DIC algorithm divides the monitored region into multiple sub-regions containing many pixel points, calculates the displacement and deformation fields of each sub-region using the information before and after deformation, and correlates each sub-region with the others. Thus, the displacement and strain fields for the entire monitored area were calculated.

A photograph of the sample before the test was set as a reference for the deformation, and the coordinate transformation relationship before and after the deformation of the pixel in the neutron region during the test loading process was as formula (3)

(3) $$\left\{ \begin{array}{l} {\tilde{x}}_{cur_{i}}=x_{ref_{i}}+u_{rc}+\frac{\partial u}{\partial x_{rc}}\left(x_{ref_{i}}-x_{ref_{c}}\right)+\frac{\partial u}{\partial y_{rc}}\left(y_{ref_{j}}-y_{ref_{c}}\right)\\ {\tilde{y}}_{cur_{j}}=y_{ref_{j}}+v_{rc}+\frac{\partial v}{\partial x_{rc}}\left(x_{ref_{i}}-x_{ref_{c}}\right)+\frac{\partial v}{\partial y_{rc}}\left(y_{ref_{j}}-y_{ref_{c}}\right) \end{array}\right.\;\left(i,j\right)\in S$$

where (x_refi_, y_refj_) and (${\tilde{x}}_{\text{curi}}$, ${\tilde{y}}_{\text{curj}}$) are the coordinates of the sub-regions before and after deformation, respectively, and (x_refc_, y_refc_) are the coordinates of the center position of the neutron region of the reference image at the initial time.

The transformation relationship between the different coordinates of the reference image before and after the deformation of the reference sub-region is as formula (4)

(4) $$\left\{ \begin{array}{l} {\tilde{x}}_{ref_{i}}=x_{ref_{i}}+u_{rr}+\frac{\partial u}{\partial x_{rr}}\left(x_{ref_{i}}-x_{ref_{c}}\right)+\frac{\partial u}{\partial y_{rr}}\left(y_{ref_{j}}-y_{ref_{c}}\right)\\ {\tilde{y}}_{ref_{j}}=y_{ref_{j}}+v_{rr}+\frac{\partial v}{\partial x_{rr}}\left(x_{ref_{i}}-x_{ref_{c}}\right)+\frac{\partial v}{\partial y_{rr}}\left(y_{ref_{i}}-y_{ref_{c}}\right) \end{array}\right.\;\left(i,j\right)\in S$$

The location of the sub-region after deformation is estimated by solving the extreme value of the correlation function [23], as formula (5):

(5) $$C_{cc}=\frac{\sum_{\left(i,j\right)\in S}\left(f\left({\tilde{x}}_{ref_{i}},{\tilde{y}}_{ref_{j}}\right)-f_{m}\right)\left(g\left({\tilde{x}}_{cur_{i}},{\tilde{y}}_{cur_{j}}\right)-g_{m}\right)}{\sqrt{\sum_{\left(i,j\right)\in S}{\left[f\left({\tilde{x}}_{ref_{i}},{\tilde{y}}_{ref_{j}}\right)-f_{m}\right]}^{2}\sum_{\left(i,j\right)\in S}{\left[g\left({\tilde{x}}_{cur_{i}},{\tilde{y}}_{cur_{j}}\right)-g_{m}\right]}^{2}}}$$

f_m_ and g_m_ are the average gray values of the reference and current images, respectively, and are expressed as the following formula (6):

(6) $$\left\{ \begin{array}{l} f_{m}=\frac{\sum_{\left(i,j\right)\in S}\left({\tilde{x}}_{ref_{i}},{\tilde{y}}_{ref_{j}}\right)}{n\left(S\right)}\\ g_{m}=\frac{\sum_{\left(i,j\right)\in S}g\left({\tilde{x}}_{cur_{i}},{\tilde{y}}_{cur_{j}}\right)}{n\left(S\right)} \end{array}\right.$$

The next step is to use a nonlinear optimizer to refine these results at a subpixel resolution by seeking the minimum expression [21], as formula (7):

(7) $$C_{LS}=\sum_{\left(i,j\right)\in S}{\left[\frac{f\left({\tilde{x}}_{ref_{i}},{\tilde{y}}_{ref_{j}}\right)-f_{m}}{\sqrt{\sum_{\left(i,j\right)\in S}{\left[g\left({\tilde{x}}_{ref_{i}},{\tilde{y}}_{ref_{j}}\right)-f_{m}\right]}^{2}}}-\frac{g\left({\tilde{x}}_{cur_{i}},{\tilde{y}}_{cur_{j}}\right)-g_{m}}{\sqrt{\sum_{\left(i,j\right)\in S}{\left[g\left({\tilde{x}}_{cur_{i}},{\tilde{y}}_{cur_{j}}\right)-g_{m}\right]}^{2}}}\right]}^{2}$$

## 3 Test procedure

### 3.1 Specimen preparation

The shale, sandstone, and limestone were cut in the same volume, wherein the shale bedding was parallel.

**Fig. 2:**
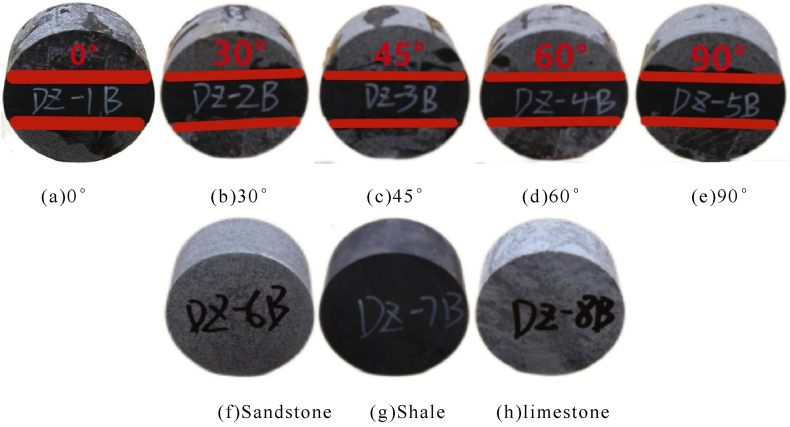
Brazilian split sample.

Sandstone, shale, and limestone were bonded to the samples and the samples were combined from top to bottom in the order of sandstone–shale–limestone. Different lithologies were bonded using an epoxy resin adhesive. The resulting rock interface was of a strong cementation type with high bonding strength and friction coefficient. Finally, we obtained a Φ50mm × 25 mm disk. Brazilian splitting test specimens (Fig. 2) were prepared using the method.

### 3.2 Test instrument

**Fig. 3:**
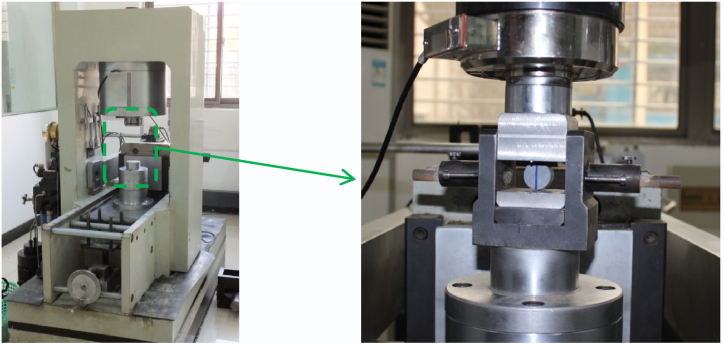
RMT-150c rock mechanics test system(Wuhan Institute of Rock and Soil Mechanics, Chinese Academy of Sciences).

Rock tensile strength tests were conducted on an RMT-150C rock mechanics test machine at the Wuhan Institute of Rock and Soil Mechanics, Chinese Academy of Sciences, as shown in Fig. 3.

Owing to the different mechanical properties of the rock, the displacement control mode was adopted in the uniaxial compression and Brazilian splitting tests at 0.002 mm/s loading rate.

**Fig. 4:**
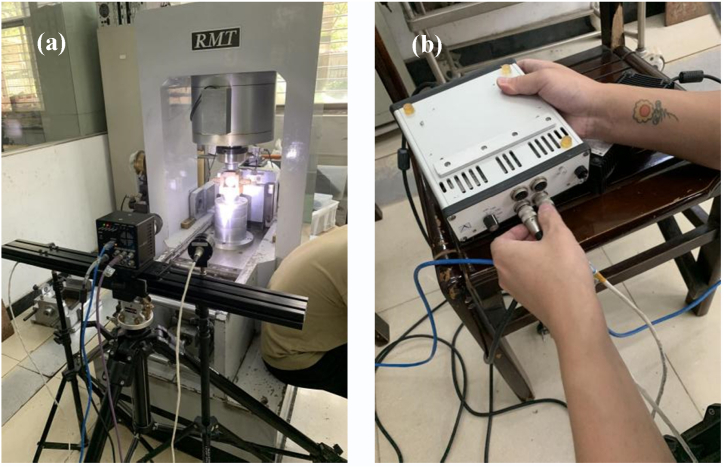
DIC system (a) High-speed cameras and (b) Image transmission systems.

The DIC test system included a camera, light source, data acquisition system, and DIC control software, as shown in Fig. 4(a and b). In this study, two GT3400 high-speed cameras (resolution: 3384 × 2074, focal length: 80 mm) were used for real-time shooting and recording sample displacement information during the test. The acquisition rate of the high-speed camera was 50 frames per second.

### 3.3 Mineral constituent

**Fig. 5:**
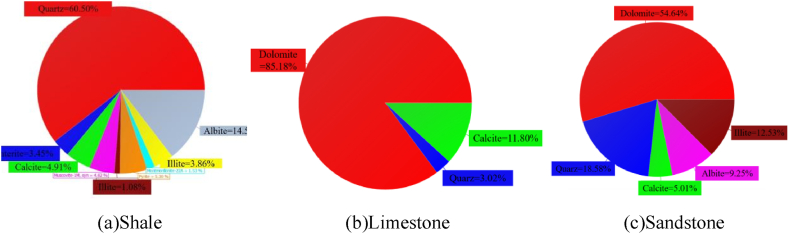
Pie chart of mineral composition of different rocks.

XRD test is carried out to analyze the mechanical properties of rocks through the mineral composition of rocks. The test results were shown in Fig. 5(a–c) and Table 1.

**Table 1:** Rock and mineral analysis results.

Lithology	Mineral content/%								
	Dolomite	Quartz	Albite	Calcite	Illite	Clino -chlore	Montmoril -lonite	Cristobalite	Pyrite
Sandstone	0	54.64	18.58	5.01	12.53	9.25	0	0	0
Shale	8.27	60.50	14.52	4.91	3.86	0	1.53	1.08	5.30
Limestone	11.80	3.02	0	85.18	0	0	0	0	0

**Fig. 6:**
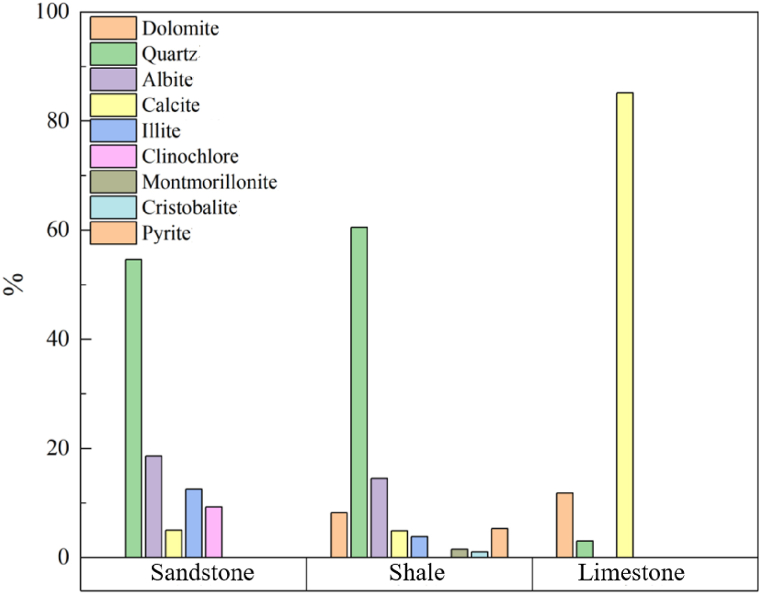
Histogram of mineral composition of different rocks.

As can be seen from the test results in Fig. 6, shale has the highest content of quartz and less content of clay minerals, and the compressive strength of shale is higher than that of the other two kinds of rocks. In contrast, the quartz content of limestone is very small, and the low-strength illite occupies the majority of the proportion.

### 3.4 Microstructure

**Fig. 7:**
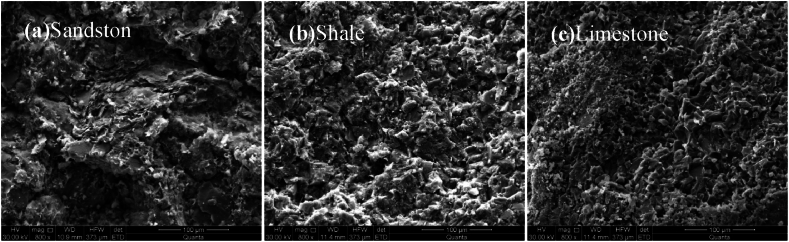
Microstructures of different rocks.

By scanning electron microscopy (SEM), it can be seen from Fig. 7(a–c) that the microstructure of shale is compact and uniform, and the adhesion between particles is good. However, the limestone presents a relatively loose particle state, and the adhesion between particles is poor. The microstructure of sandstone is relatively dense, but the particle distribution is not uniform, which affects its mechanical properties.

### 3.5 Test results and analysis

Table 2 shows the brazilian splitting test results.

**Table 2:** Brazilian splitting test results.

Serial number	Angle	Height/mm	Diameter/mm	Weight/g	Ultimate load/KN	Strength of extension/MPa
DZ-1B	0°	25.21	50.12	125.53	7.56	3.81
DZ-2B	30°	25.11	50.24	125.42	7.03	3.55
DZ-3B	45°	25.22	50.09	125.36	7.89	3.98
DZ-4B	60°	25.09	50.11	125.14	7.23	3.66
DZ-5B	90°	25.14	50.21	125.64	7.13	3.59
DZ-6B	Sandstone	25.16	50.31	125.40	6.07	3.05
DZ-7B	Shale	25.22	50.08	125.04	6.90	3.47
DZ-8B	Limestone	25.03	50.11	132.85	8.23	4.18

**Fig. 8:**
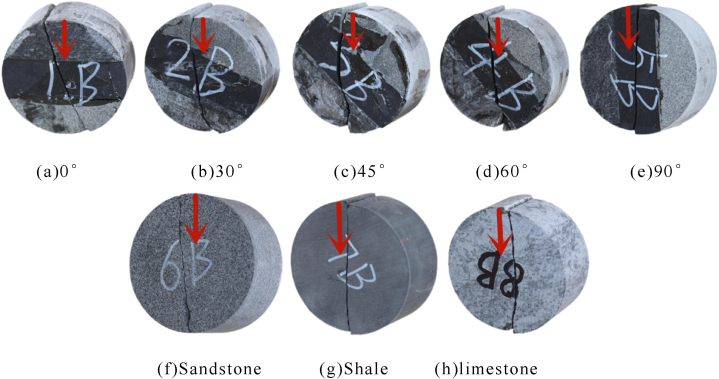
Failure and force-displacement diagram of Brazilian splitting test.

Since the tensile strength of the rock is relatively low and the strength of epoxy resin-bonded rock interface is relatively high, the fracture morphology of rock assembly and unilithologic rocks are not significantly different during Brazilian splitting tests at 0°, 30°, and 45°. However, Fig. 8 indicates that with an increase in the interface angle, the split crack developed from a linear crack to an arc-shaped crack. At 60°, when the fracture expanded to the shale–limestone interface, a certain deviation occurs along the interface; the reasons are similar to those of the previous results of the compressive strength of rock assembly. The surface roughness of sandstone was significantly higher than that of limestone, and the interfacial strength of sandstone and shale increased when bonded. The tensile strength of rock mass at 90° was similar to that of single shale.

**Fig. 9:**
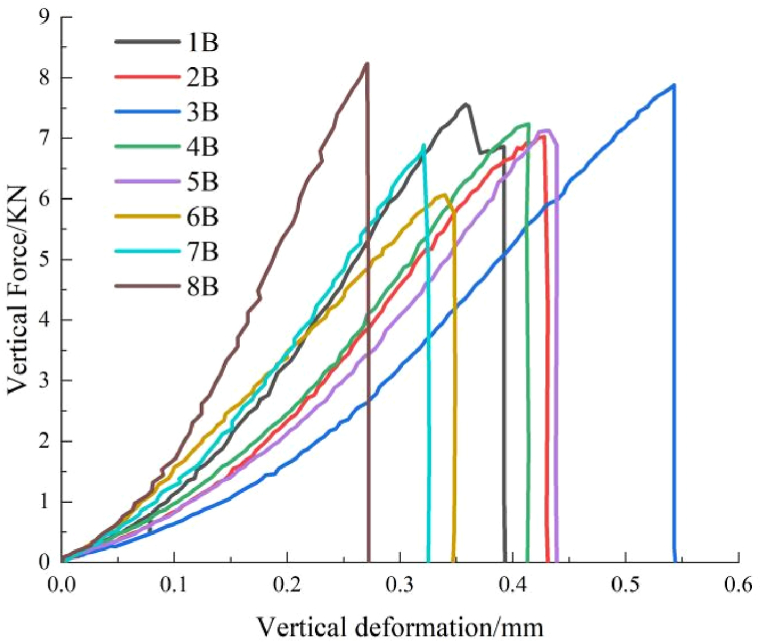
Failure and force-displacement diagram of Brazil splitting test.

Fig. 9 is a breakdown and force-displacement diagram of the Brazilian splitting test.

**Fig. 10:**
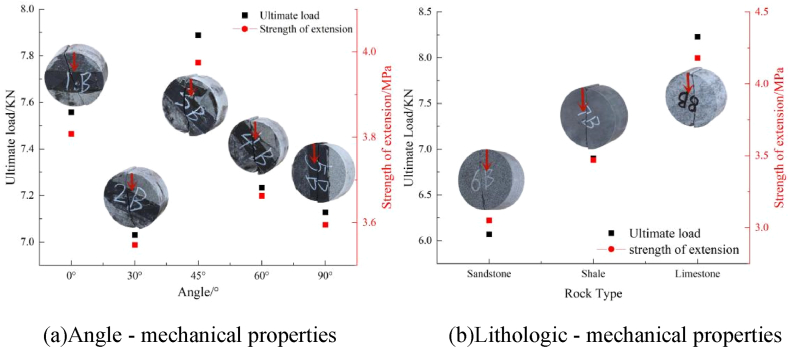
Diagram of angular and lithologic mechanical properties.

The ultimate load and tensile strength of rock assemblage in the Brazilian splitting test initially decreased, then increased, and further increased with an increase in the angle (Fig. 10(a)). Moreover, limestone had the highest tensile strength, followed by shale and sandstone (Fig. 10(b)).

## 4 DIC-based study on brazilian split fracture propagation

**Fig. 11:**
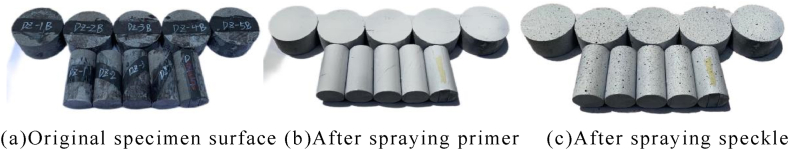
Speckle making process of digital image correlation (DIC) sample.

The rock mass comprises three rocks with different lithologies and exhibits strong heterogeneity. Owing to the differences in lithology, the interface properties of different rocks are different; therefore, determining the failure mode of a rock mass using a simple rock mechanics test is difficult. DIC is a non-destructive, real-time, efficient, and full-strain-field surface deformation monitoring technique that addresses the problem that traditional deformation monitoring methods cannot perform at fine scales during testing. Prior to the test, a speckle was created on the sample surface (Fig. 11) to calculate the displacement and strain fields of the specimen surface by monitoring the deformation information of the speckle during the test.

### 4.1 Analysis at 0°

**Fig. 12:**
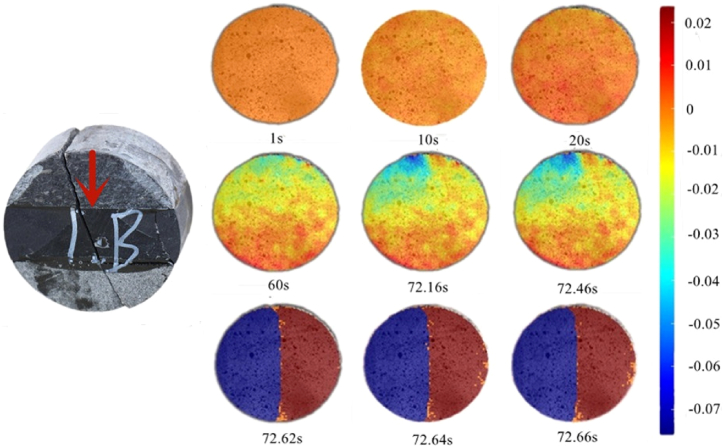
Displacement nephogram in X-direction (0°).

The displacement nephogram in the X-direction (Fig. 12) shows that rock mass deformation was relatively uniform from 1 to 30 s. Rock mass deformation in all directions was almost consistent on rock sample surface, and the entire rock mass was in interfacial deformation. Owing to the difference in rock deformation ability, the rocks deformed unevenly after the interface deformation stage and entered the elastoplastic stage, as shown in the nephogram for 60 s. After 60 s, the deformation of the rock assembly in the X-direction tended to be stable and the deformation of the limestone rock sample and the interface between limestone and shale was significantly higher than that of sandstone. At 72.62 s, rock fracture occurred in the middle.

Through DIC image analysis, it can be concluded that the Brazilian splitting test of the rock assembly can be divided into three stages: interface deformation, elastoplastic, and failure.

Combined with the X-direction nephogram, Fig. 12 shows that 1–20 s indicates the interface deformation stage of the rock sample. At this stage, the overall rock sample deformation was relatively uniform, and the maximum principal strain gradually developed until it became prominent at 60s. At 72.16 s, the maximum principal strain appeared in the middle area of the top of the rock sample and continuously moved downward. At 72.62 s, the rock samples were fractured.

**Fig. 13:**
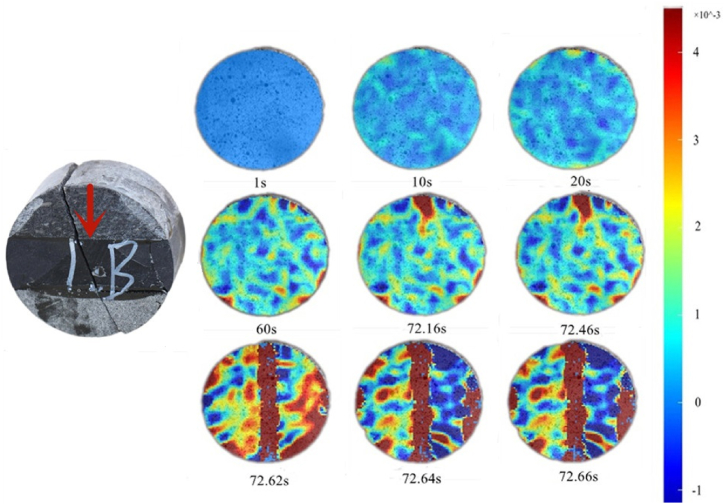
Maximum principal strain nephogram (0°).

The nephogram of the maximum principal strain (as shown in Fig. 13) shows that when the maximum principal strain develops downward close to the middle of the rock sample, rock failure occurs. This is because when the Brazilian disk was subjected to a concentrated load at the top, the interior of the rock mainly exhibited tensile stress, and the greatest tension was felt at the center of the disk. Therefore, when the maximum principal strain developed close to the middle of the rock sample, the rock lost its bearing capacity, resulting in failure.

### 4.2 Analysis at 30°

**Fig. 14:**
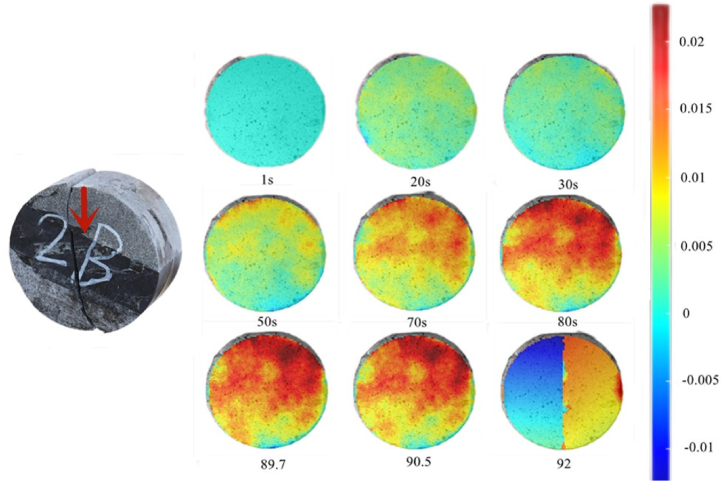
X-direction displacement nephogram (30°).

This is similar to the X-direction displacement nephogram of the Brazilian disk at 0°, and as shown in Fig. 14, the interface deformation stage was 1–30 s at 30°. The X-direction displacement of the disk in all directions was essentially the same. The rock entered the elastic–plastic stage after 30s. Different from that at 0°, the sandstone and shale substantially deformed after 50 s at 30°. This is because at 0°, pressure is the only force on the interfacial caulk (epoxy resin) and the rock body. At 30°, the interfacial caulk (epoxy resin) and the rock body are subjected to not only pressure, but also shear force; the interfacial filler (epoxy resin) is a plastic material, which will have a displacement relative to the rock (brittle material) under the action of shear force along the interfacial direction. Therefore, at 30°, the rock mass in the sandstone and shale parts will deform considerably in the X-direction. The elastoplastic phase lasted for 90.5 s, followed by failure at 92 s.

**Fig. 15:**
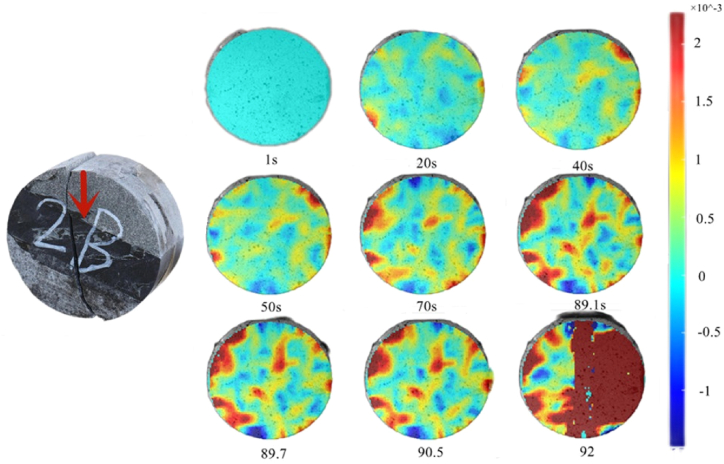
Maximum principal strain nephogram (30°).

From Fig. 15, at 1–40 s, the rock is in the interfacial deformation stage, and the maximum principal strain is prominent at 50 s. However, the maximum principal strain begins to develop at the junction of sandstone and shale, rather than at the top of the rock sample assembly due to the action of the interface. Subsequently, the maximum principal strain manifested in the middle of the rock assemblage at 70 s under the action of tensile stress, and the salient region expanded at 89.1 s. At 89.7 s, the maximum principal strain developed stably in the middle region of the rock assemblage and gradually shifted along the interface. Finally, the rock mass broke in the middle at 92 s.

### 4.3 Analysis at 45°

**Fig. 16:**
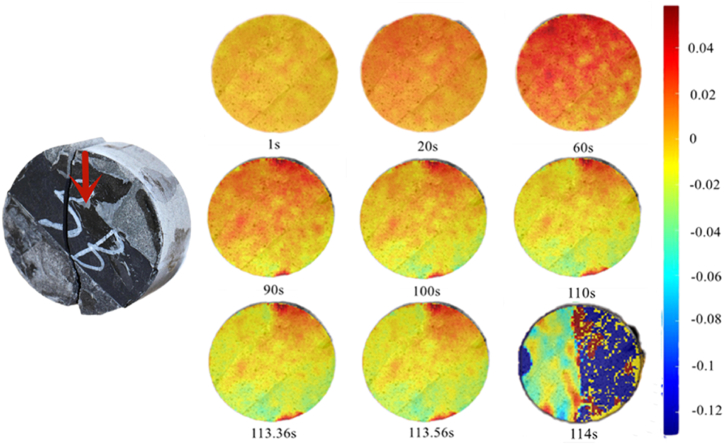
X-direction displacement nephogram (45°).

According to the X-direction displacement nephogram at 45°,as shown in Fig. 16, rock sample assembly was in the interface deformation stage during 0–60 s and deformed in the sandstone part at 90 s; however, the sandstone part had different deformation forms on the left and right sides. The left side deformed evenly at 45°, whereas the right side showed stress concentration because of its proximity to the loading point and the interface. The loading point acts on the boundary of the interface, which has a stronger plastic condition than the rock. After 110 s, the overall rock mass deformation decreased, energy accumulated in the rock mass body, and brittle failure occurred at 114 s.

**Fig. 17:**
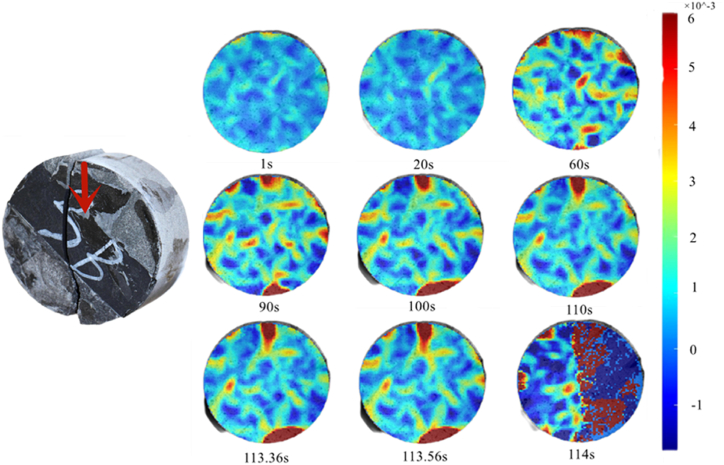
Maximum principal strain nephogram (45°).

The sandstone and limestone parts of the rock assemblage showed obvious strain at 90 s and the strain of the limestone part transversed, mainly owing to the effect of pressure as shown in Fig. 17. The stress area of sandstone at 45° was smaller than those at 0° and 30°, and the interface boundary was close to the loading point. Moreover, sandstone is significantly higher more brittle than the two other rock types. Therefore, the sandstone part moved downward and the principal strain deflected when it met the interface. Finally, the failure of the rock mass occurred at 114 s.

### 4.4 Analysis at 60°

**Fig. 18:**
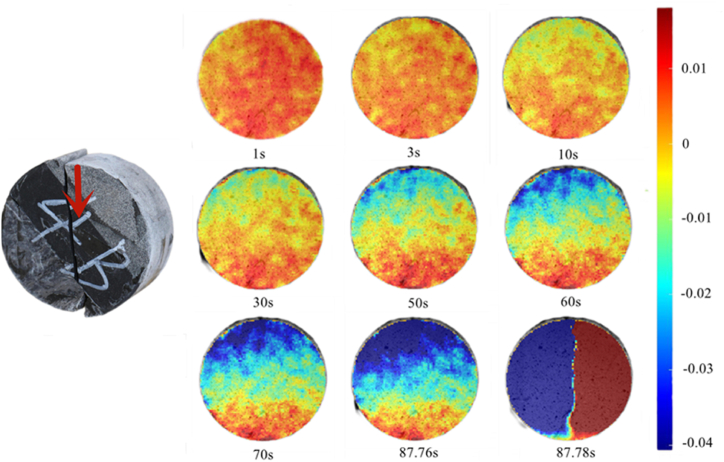
X-direction displacement nephogram (60°).

Interface deformation stage occurred during 1–10s as shown in Fig. 18 and the deformation in the X-direction tended to be stable. After 30 s, the deformation area progressed stepwise. Initially, the deformation ranges of the three rocks were similar. After 50 s, the top sandstone was almost undeformed, while the deformation range of the central shale was slightly larger than that of the sandstone and that of the limestone is the largest. Over time, the deformation area gradually decreased until only a tiny part of the limestone was finally deformed. Uniaxial tests showed that the fracture occurred at the lower interface of the cylindrical rock mass at 60°; therefore, it is reasonable to explain the high deformation of limestone rocks during the Brazilian splitting test at 60° and the rock assembly finally broke at 87.78 s.

**Fig. 19:**
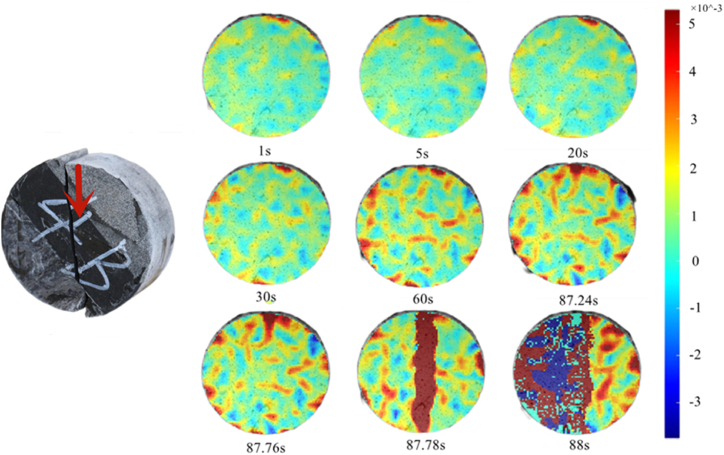
Maximum principal strain nephogram (60°).

The maximum principal strain nephogram at 60° as shown in Fig. 19 indicates that the maximum principal strain appeared at the top of the sandstone at the end of the interface deformation stage (20 s),and gradually developed within the specimen with the progress of the test.

At 87.24 s, the main strain in the central part of the rock mass expands downward along the interface under the influence of the interface. Finally, the rock mass fractured at 87.78 s.

### 4.5 Analysis at 90°

**Fig. 20:**
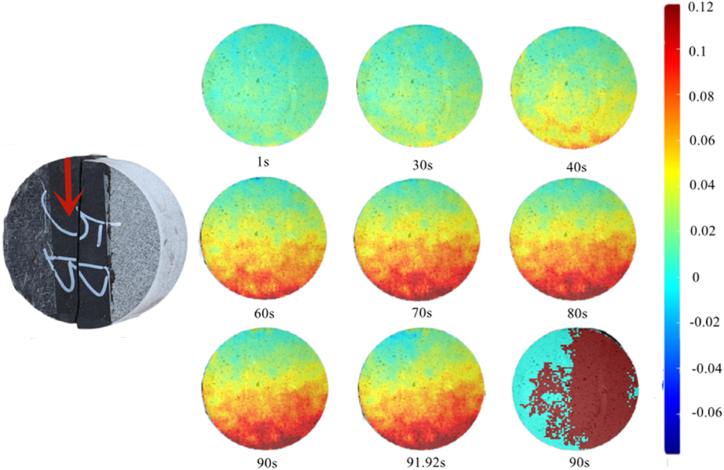
X-direction displacement nephogram (90°).

At 90°, the main stress area was the shale part, which had a high degree of compactas as shown in Fig. 20. Therefore, the rock assembly had no obvious interface deformation stage. In the elastic–plastic stage, the displacement in the X-direction was primarily concentrated at the bottom, and the rock assembly fractured at 92.76 s.

**Fig. 21:**
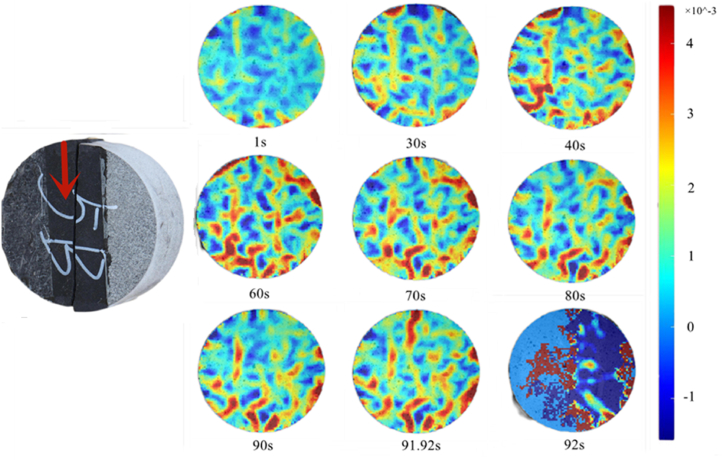
Maximum principal strain nephogram (90°).

Combined with the displacement nephogram at 90°, the maximum principal strain of rock sample at the elastic–plastic stage developed stably as shown in Fig. 21, which was different from that at 0°, 30°, 45°, and 60°. At 90°, the maximum principal strain is highlighted below the rock sample rather than above it first. Moreover, at 91.92 s, the maximum principal strain is prominent above the rock sample and shifts downward.

**Fig. 22:**
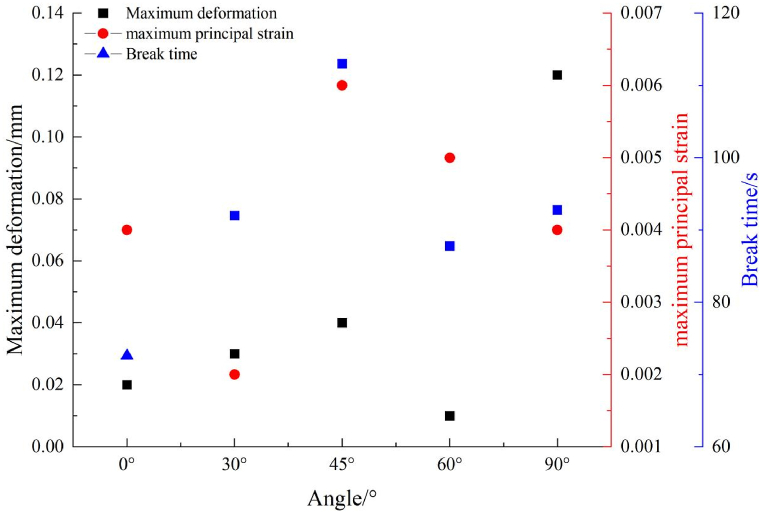
Maximum deformation and maximum principal strain at different angles.

The variation of the maximum principal strain with angle is consistent with the variation trend of the tensile strength with the angle, which decreases, increases, and then decreases as shown in Fig. 22.

The variation of rupture time is reverse to that of the maximum principal strain, the rupture time at 45°, 0°, and 30° is the longest, shortest, close to that at 90°, respectively.

## 5 Discussion

### 5.1 Influence of multilithology on hydraulic fracture propagation

**Fig. 23:**
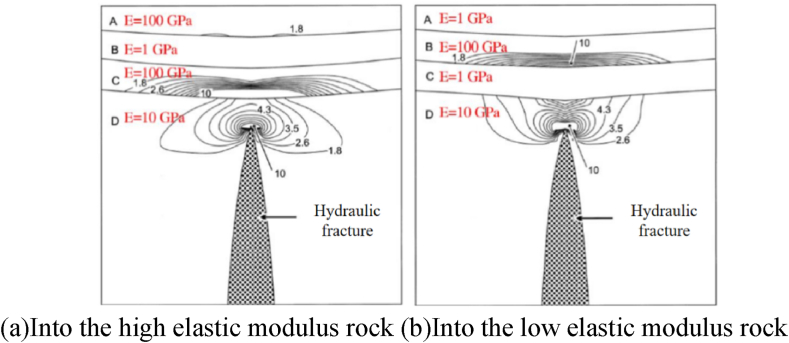
Distribution of tensile stress caused by fractures in strata with different modulus [24].

During reservoir reconstruction by hydraulic fracturing, it is inevitable to encounter multi-lithologic geological environments, and the differences in mechanical properties between different rocks introduce more uncertainties to fracturing and affect the propagation law of hydraulic fractures. Therefore, studying the mechanical characteristics of multi-lithologic rocks has important significance for reservoir fracturing. During hydraulic fracturing, the propagation of fractured multi-lithologic reservoirs is affected by interfacial action. If the hydraulic fracture is captured by the bedding plane, the complexity of the hydraulic fracture network is significantly reduced, and horizontal well fracturing can prevent the influence of bedding on hydraulic fracture propagation. Moreover, when the rock layer with a higher elastic modulus penetrates the rock layer with a lower elastic modulus during hydraulic fracture, the interfracture stress intensity factor is reduced, thus preventing the propagation of hydraulic fractures in the rock layer with a lower elastic modulus. According to Gudmundsson [24], when a hydraulic fracture approaches a rock with high elastic modulus from a rock with low elastic modulus, the tensile stress at the crack tip causes stress concentration at the bottom of the rock with high elastic modulus (Fig. 23); consequently, the tensile stress increases and the fracture can be extended; otherwise, it will hinder the fracture expansion, which in turn affects the expansion of hydraulic fractures to adjacent rock strata; therefore, the hydraulic fracture area is elliptical and hydraulic fracturing reduces the area of reservoir reconstruction.

### 5.2 Brazilian split fracture interface characteristics

During hydraulic fracturing, fracturing failure mainly occurs in rocks. Therefore, studying the fracture characteristics of rock assemblages after fracturing failure can provide a reference for evaluating the reservoir fracture-building ability [25].

Geotechnical engineering uses image-processing methods to identify and detect cracks to evaluate their characteristics. Based on the fractal theory proposed by Mandelbrot [23], this study adopted the fractal dimension calculation method for a three-dimensional cracked square box [26]. The fractal dimensions were calculated using the following formula(8):

(8) $$D=\lim_{\delta_{i}\to 0}\frac{\log\left(N\left(\delta_{i}\right)\right)}{\log\left(\delta_{i}\right)}$$

D, Fractal dimension; δ_i_, The ith time square box count size; N, Number of square boxes. Note: The fractal dimension is a double logarithmic fitting slope.

**Fig. 24:**
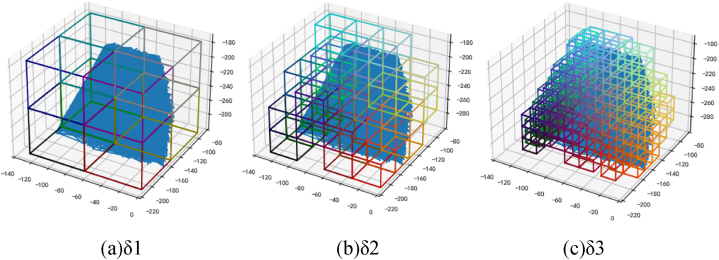
Fractal dimension calculation method of three-dimensional cracked square box.

The calculation process of fractal dimension D was shown in Fig. 24(a–c).

**Fig. 25:**
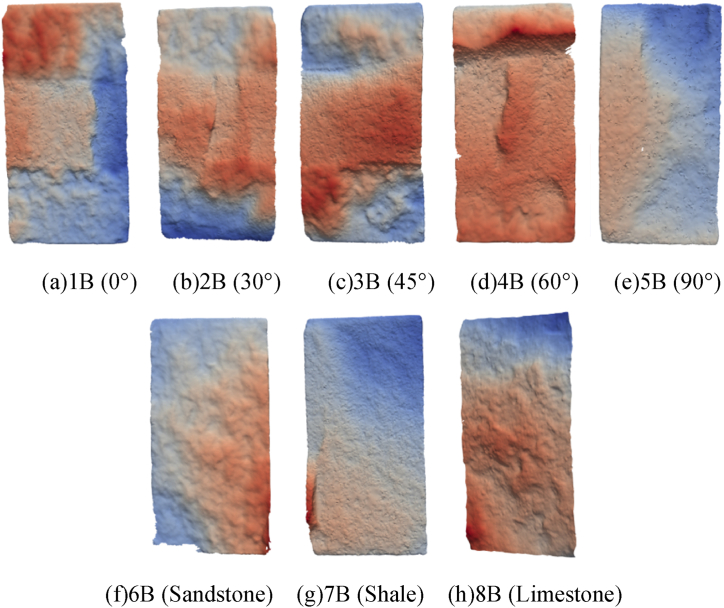
Fracture surface characteristics.

First, the crack surface was scanned and treated using a high-precision 3D topography scanner, and the fracture morphology after the split failure of the assembly was analyzed using a 3D crack square box fractal dimension calculation method. The results were as follows Fig. 25.

**Fig. 26:**
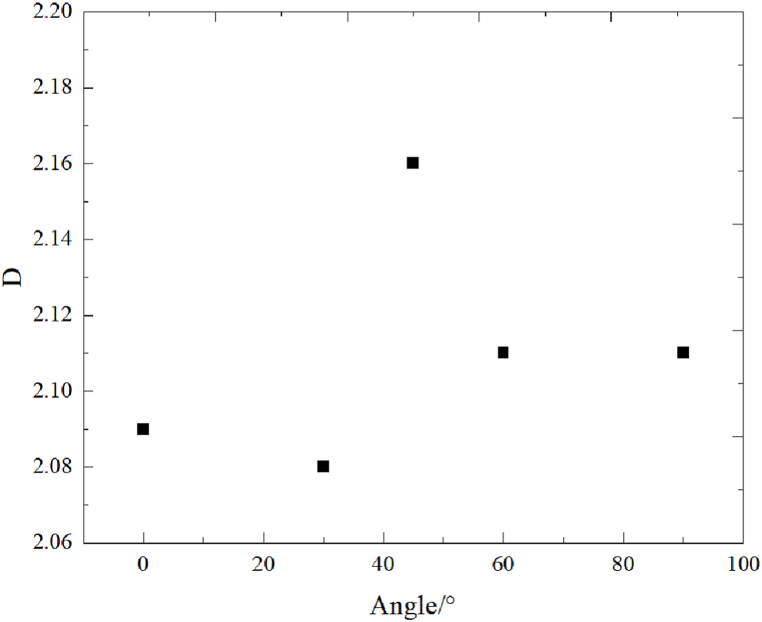
Angle–D.

(1) Fig. 25 shows that cracks in the Brazilian splitting test crossed the junction between rocks, with changes in the fluctuation degree. At 0°, the characteristics of partial fractures in sandstone were the most evident; at 45°, the characteristics of fractures across the interface were the most evident; at 60°, the fluctuation degree of fracture surface was uniform; at 90°, the fracture characteristics were basically consistent with those of single shale.
(2) From Fig. 26, the fractal dimension D first decreased, then increased, and further decreased with an increase in angle, which is the same as the variation of the ultimate load in the Brazilian splitting test and the maximum principal strain in the DIC test, indicating that crack roughness is related to ultimate load and maximum principal strain. Although the law of change can be found, it cannot be analyzed in combination with mechanical principles. As mentioned above, in the Brazilian splitting test, all cracks in sample 5B (angle 90°) appeared in the shale area, the ultimate load of sample 5B was close to that of sample 7B (only shale lithology), and the fractal dimension was the same, indicating that the two had the same roughness.

## 6 Conclusion

The multi-lithology characteristics of the reservoir make its mechanical properties complicated and its failure mechanism unclear, which causes confusion for the field work. In this study Brazilian splitting, and DIC tests were conducted on sandstone, shale, and limestone assemblages. The following conclusions were reached.

(1) The Brazilian splitting test shows that the ultimate load and tensile strength of the rock mass first decrease and then increase with an increase in angle. During the Brazilian splitting test of rock assembly at 0°, 30°, and 45°, there was no obvious difference between the fracture morphology of rock assembly and unilithologic rock were not substantially different. As the interface angle increased, the fracture developed from a linear to an arc fracture. At 60°, when the fracture extended to the shale–limestone interface, there was a certain deviation along the interface at the interface. At 90°, the tensile strength of the rock assembly was almost the same as that of a single shale.
(2) The DIC test shows that the rock assembly can be divided into three stages during the Brazilian splitting test: interface deformation, elastoplastic, and failure. At 0° and 90°, the failure characteristics are similar to those of a unilithologic rock; however, affected by the difference in rock mechanical properties, the deformation in the X-direction is uneven at the interface deformation stage at 0° and 90°, while the influence on the maximum principal strain is not obvious. At 30°, 45°, and 60°, the angle of the interface not only affects the deformation in the X-direction but also causes the development of maximum principal strain toward the interface.
(3) With an increase in angle, the fractal dimension D first decreased, then increased, and then decreased, which was the same as the variation trend of the ultimate load in the Brazilian splitting test and the maximum principal strain in the DIC test with angle.
(4) With the increase of the Angle, the fractal dimension D showed a trend of first decreasing, then increasing and then decreasing, which was the same as that of the ultimate load in the Brazilian splitting test and the maximum principal strain in the DIC test.

This study can provide valuable insights into the process and characteristics of fracture propagation during field hydraulic fracturing operations. By offering a deeper understanding of how fractures develop and interact within the reservoir rock, it serves as a useful reference for optimizing fracturing designs, improving operational efficiency, and ultimately enhancing the recovery of hydrocarbon resources.

## CRediT authorship contribution statement

**Bo Zeng:** Conceptualization. **Guozhou Qiu:** Funding acquisition, Formal analysis, Conceptualization. **Haoyong Huang:** Data curation. **Yintong Guo:** Formal analysis. **Ersi Xu:** Investigation. **Junchuan Gui:** Methodology. **Junfeng Li:** Resources.

## Data availability statement

Data will be made available on request.

## Declaration of competing interest

The authors declare that they have no known competing financial interests or personal relationships that could have appeared to influence the work reported in this paper.
